# Effect of laser etching on shear bond strength between polyether ether ketone (PEEK) and maxillofacial silicone after accelerated ageing – an *in vitro* study

**DOI:** 10.2340/biid.v12.45066

**Published:** 2025-12-17

**Authors:** Ganesh RamKumar Rajapandi, Ahila Singaravel Chidambaranathan, MuthuKumar Balasubramanium

**Affiliations:** Department of Prosthodontics, SRM Dental College, Ramapuram, Bharathi Salai, Chennai-89, Tamil Nadu, India

**Keywords:** Laser, maxillofacial prostheses, silicone, PEEK

## Abstract

**Introduction:**

Debonding of silicone prostheses from metal substructures is a frequently reported complication in prosthodontics. Polyether ether ketone (PEEK) has emerged as a promising alternative framework material owing to its favourable biomechanical properties; however, its limited bond strength to silicone remains a concern. The present study aimed to evaluate the shear bond strength between PEEK and maxillofacial silicone following laser etching of PEEK and subsequent accelerated ageing.

**Materials and methods:**

According to ISO 10477:2020, 128 PEEK specimens were fabricated using Computer-Aided Design and Computer-Aided Manufacturing (CAD/CAM) with 10 mm diameter and 3 mm thickness, and silicone specimens with 5 mm diameter and 2.5 mm height. The specimens were categorised into: Group 1–no surface treatment, Group 2–Al_2_O_3_ air abrasion, Group 3–98% sulphuric acid etching, and Group 4–laser irradiation. The silicone specimens were bonded to PEEK and kept at room temperature for 24 h for polymerisation, and were subjected to accelerated ageing for 252, 504, and 1,008 h. The shear bond strength was evaluated using universal testing machine at 1 mm/min crosshead speed. The comparison within groups was done using one-way analysis of variance (ANOVA) and multiple group comparison was done using Tukey’s HSD (Honestly Significant Difference) post-hoc analysis.

**Results:**

Statistical analysis showed that surface pretreatment had a significant effect on bond strength (*p* < 0.05). Laser treatment and air abrasion produced significantly higher bond strengths compared to sulfuric acid etching, while no significant difference was found between laser treatment and air abrasion. Accelerated ageing time also had a significant influence, with bond strength values decreasing progressively from 252 h to 1,008 h across all pretreatment groups.

**Conclusion:**

Surface pretreatment significantly influenced the adhesion of maxillofacial silicone to PEEK. Laser treatment and air abrasion provided superior and statistically comparable bond strengths, whereas sulfuric acid etching was less effective. Accelerated ageing reduced bond strength over time, highlighting the effect of ageing conditions on the durability of adhesion.

## Introduction

Skeletal structure influences the appearance and function of the human body. Any bony abnormalities or deficiency make it challenging for a person to lead a relatively normal life. Maxillofacial prostheses will restore the function and aesthetics of the lost area [1]. Extraoral prostheses are usually fabricated with acrylic resin or maxillofacial silicone, which were retained by several methods such as osseo-integrated implants, adhesives, body cavities, spectacles, and teeth [2]. Silicones are widely used due to their good biomechanical properties, while acrylic resins are commonly used as substructure framework in extra oral prostheses [2, 3]. Debonding of silicone prostheses from the metal substructure is the frequently reported problem in prosthodontics. Polyether Ether Ketone (PEEK) is an alternative option for framework fabrication of any dental prosthesis which is a polymer and needs to achieve good bond strength for the prosthesis’s durability. Maxillofacial prostheses substructure can be constructed by using PEEK due to the advantages of elimination of allergies and metallic taste, low affinity to plaque accumulation, and excellent wear resistance [4].

The high-performance PEEK has recently become significant in dentistry because of its high temperature stability and excellent biomechanical properties. PEEK are widely used in the fabrication of dental implants, implant retained bars, temporary abutments, and frameworks for removable prostheses. The material possesses excellent biomechanical properties, X-ray transparency, comparable elastic modulus to human bone, and serves as an effective alternative for individuals allergic to metal prostheses [5].

The disadvantage of PEEK is that since it has an inert surface and a low surface energy, it is difficult to get good bond strength to other materials. To increase the shear bond strength of PEEK with other materials, surface treatment has been employed for years together. Physical surface modification methods include plasma and ultraviolet (UV) radiation. In contrast, chemical methods involved processes such as acid etching, oxidation, and chemisorption. In addition, radiation-based techniques comprise plasma gas, gamma irradiation, ion beam, electron beam, and lasers with varying wavelengths. The topography of the PEEK gets treated by surface modification by physical or chemical methods, which modify at atomic and molecular level [6, 7].

Lasers are photons, which are extremely energetic and intense particles. Some lasers induce crosslinking and chain scission on the surface of polymers. Its high energy causes sintering effects on surfaces. The shear bond strength between PEEK and composite resins has been shown to improve by laser surface treatments, but there is no research available on bond strength between PEEK and maxillofacial silicone after laser surface treatment [6]. Hence, this study was conducted with the objective of evaluating the effect of laser irradiation on shear bond strength between PEEK and maxillofacial silicone 24 h after fabrication, and after 252, 504, and 1,008 h of accelerated ageing. It was hypothesised that surface treatment of PEEK using laser irradiation would not result in higher shear bond strength compared to air abrasion with Al₂O₃ and 98% sulfuric acid etching. Furthermore, it was expected that the durability of the bond would remain unchanged across different surface treatments when subjected to accelerated ageing conditions at 252, 504, and 1,008 h.

## Material and methods

### Sample size estimation and specimen distribution

This research was approved by the Institutional Review Board of SRM Dental College, Ramapuram, Chennai, TamilNadu, India. (SRMDC/IRB/2022/MDS/No.203). The sample size was calculated using G*Power software (version 3.1.9.2; Heinrich-Heine University). The power of the study was 95% and 5% alpha error, and the Effect size *f* = 0.35. The total sample size was 128, and the specimens were categorised into four main groups based on the type of surface treatment. Each main group comprised 32 specimens (*n* = 32), which were further subdivided into subgroups of eight specimens each (n = 8) according to the ageing hours. The specimens were categorised into Group 1–no surface treatment, Group 2–Al_2_O_3_ air abrasion, Group 3–98% sulphuric acid etching, and Group 4-laser irradiation. The silicone specimens were bonded to PEEK and kept at room temperature for 24 h for polymerisation, and were subjected to accelerated ageing for 252, 504, and 1,008 h.

### Specimen preparation

**Figure 1:**
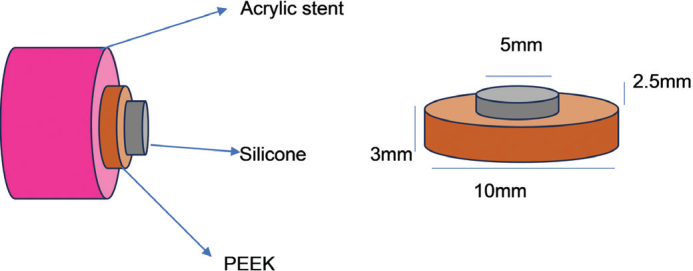
Schematic representation of specimens.

According to ISO 10477:2020 [8], a total of 128 PEEK specimens were made by using CAD/CAM (In Lab MC X5, Dentsply Sirona, Bensheim, Germany) with 10 mm diameter and 3 mm thickness (Figure 1). The finishing of the PEEK specimen was done with 1200 Grit carbide paper (3M India Limited, Bangalore, Karnataka, India). The specimen was rinsed in running water for 10 s, and was kept in an ultrasonic cleaner (GT Sonic Cleaner, Waldent, New Delhi, India) for 5 min.

### Surface treatment

Group 1 PEEK specimens had no surface treatment. Group 2 PEEK specimens were treated with airborne abrasion using 110 μm aluminium oxide particles (Al₂O₃) (Alminox, Delta, Chennai, India) at 2 bar pressure from a distance of 15 mm for 15 s [9]. Group 3 PEEK specimens were treated with 98% (H_2_SO_4_) concentrated sulfuric acid (Chenchem Chemicals, Chennai, India) for 60 s, followed by rinsing with distilled water for 1 min and drying with oil-free compressed air [10]. Group 4 PEEK specimens were surface treated using a Yb: PL laser with a power setting of 5 W and a frequency of 250 ms (Meera Laser Solution, Chennai, Tamil Nadu, India). The laser was applied in non-contact mode at a fixed working distance of 17.8 mm to achieve surface etching [9].

### Silicone specimen preparation

A ring-shaped die was fabricated using fused deposition modelling (FDM) 3D printing method (Bambu Lab, Shenzhen, China), with an outer diameter of 10 mm, an inner diameter of 5 mm, and a height of 2.5 mm, to retain the silicone during attachment to the PEEK surface.

Then the room-temperature vulcanising maxillofacial silicone (RTV) (M511, Technovent, Bridgend, UK) was mixed with a ratio of 10:1 (5.650 g base:0.5 g catalyst) using a spatula for 3–4 min and the silicone was packed into the 3D printed ring shaped die with 2.5 bar pressure with parallel force towards PEEK specimens and was allowed to set for 8 h at room temperature [11]. Thereafter, the specimens were kept under dry conditions for 24 h, without accelerated ageing. The specimens were then tested for shear bond strength and followed by accelerated ageing for 252, 504, and 1,008 h.

### Ageing procedure

The specimens were then placed in a xenon test chamber (Q SUN XE 2, West Lake, Ohio, USA) for 102 min of (29°C ± 2°C) dry weather cycle and 18 min of (36°C ± 2°C) wet weather cycle with controlled flow of distilled water, The humidity was maintained at 70% and the air pressure at 700–1,060 hpa. The xenon light (150 Klx) was applied for accelerated ageing for 252, 504, and 1,008 h [12].

### Shear bond strength test

After accelerated ageing, the samples were subjected to shear bond strength testing. To hold the specimens in position in a universal testing machine during testing, an acrylic stent was fabricated with 20 mm diameter internally and 25 mm externally using auto polymerising polymethyl methacrylate resin (DPI, Uttarakhand, India).The auto polymerising polymethyl methacrylate resin (DPI, Uttarakhand, India) resin was mixed according to the manufacturer’s instructions with a ratio of 3:1 (polymer: monomer) and placed into the master die.

A universal testing machine (Instron, Norwood, USA) was employed to measure shear bond strength. Specimens were positioned in a custom-fabricated loading fixture, and the test was carried out at a crosshead speed of 1 mm/min. The maximum force exerted before bond failure was documented. Shear bond strength was determined using the equation BS = F/A, where F is the applied force at failure and A is the bonded surface area [13].

### Failure mode analysis

After the shear bond strength test, the type of failure was assessed using a stereo microscope (Labomed Inc, Los Angels, USA). If failure had occurred between PEEK and silicone it was considered as an adhesive failure, if the failure had occurred in the silicone, it was considered as a cohesive failure, and the failure type was classified as mixed when both adhesive and cohesive components were observed.

### Statistical analysis

The obtained shear bond strength values were subjected to statistical analysis using Statistical Package for the Social Sciences (SPSS), version 29.0.0 (IBM Corp, Armonk, NY, USA). The comparison within group was done using one-way analysis of variance (ANOVA) and multiple group comparison was done using Tukey’s HSD (Honestly Significant Difference) post-hoc analysis.

## Results

**Figure 2:**
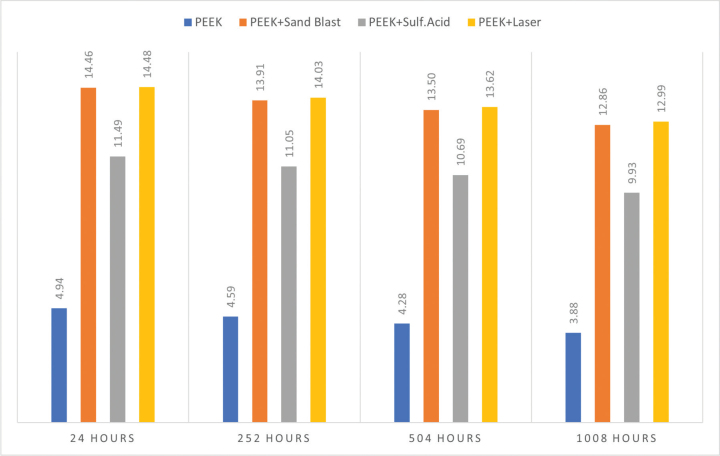
Inter group comparison of shear bond strength (MPa) between PEEK and maxillofacial silicone after 24 h of fabrication, 252 h to 504 h and 1,008 h of accelerated ageing.

The shear bond strength data are presented in Table 1 and Figure 2. The one-way ANOVA revealed that surface pretreatment had a significant effect on bond strength at all ageing intervals (p < 0.05) (Table 2). Post-hoc comparisons indicated that laser treatment and air abrasion produced significantly higher bond strengths than sulfuric acid etching and the untreated control, whereas no significant difference was found between laser treatment and air abrasion.

**Table 1:** Mean and standard deviation of shear bond strength (MPa) between PEEK and maxillofacial silicone.

Timeline	Group	Mean	SD	95% Confidence interval for mean		*P*-value
				Lower bound	Upper bound	
24 h after fabrication	Control	4.94	0.18	4.79	5.09	0.0001
	Air abrasion	14.45	0.37	14.14	14.76	
	Acid etching	11.48	0.49	11.07	11.89	
	Laser	14.48	0.52	14.04	14.91	
252 h	Control	4.58	0.25	4.37	4.79	0.0001
	Air abrasion	13.90	0.37	13.59	14.22	
	Acid etching	11.05	0.49	10.64	11.46	
	Laser	14.02	0.54	13.56	14.48	
504 h	Control	4.28	0.28	4.04	4.51	0.0001
	Air abrasion	13.50	0.40	13.16	13.84	
	Acid etching	10.69	0.44	10.32	11.06	
	Laser	13.62	0.59	13.12	14.11	
1,008 h	Control	3.87	0.22	3.69	4.06	0.0001
	Air abrasion	12.85	0.55	12.39	13.31	
	Acid etching	9.92	0.43	9.56	10.29	
	Laser	12.99	0.47	12.59	13.38	

SD: standard deviation.

**Table 2:** The comparison of shear bond strength within the group using One-way ANOVA.

Time line	Group	Sum of squares	df	Mean Square	*F*	Sig.
24 h after fabrication	Between Groups	868.37	4	217.09	1379.68	0.000
	Within Groups	5.51	35	0.157		
	Total	873.88	39			
252 h	Between Groups	844.15	4	211.04	1213.50	0.000
	Within Groups	6.09	35	0.17		
	Total	850.24	39			
504 h	Between Groups	823.93	4	205.98	1131.97	0.000
	Within Groups	6.37	35	0.18		
	Total	830.30	39			
1,008 h	Between Groups	790.76	4	197.69	1061.22	0.000
	Within Groups	6.52	35	0.19		
	Total	797.28	39			

ANOVA: analysis of variance; df: degrees of freedom.

After 24 h of sample fabrication and accelerated ageing time (252, 504, and 1,008 h), untreated PEEK consistently demonstrated the lowest bond strength, while laser treatment and air-abrasion specimens maintained significantly higher values compared to sulphuric acid-etched specimens.

Ageing time also showed a significant effect on bond strength across all pretreatments. Progressive reductions were observed with increasing ageing duration, with the difference between 252 and 1,008 h reaching statistical significance in all groups. Despite this decline in bond strength, laser treatment and air abrasion continued to outperform sulfuric acid etching throughout the study period (Table 3).

**Table 3:** The multiple group comparison using post-hoc analysis.

Time line	(I) Group	(J) Group	Mean difference (I-J)	Std. error	Sig.	95% confidence interval	
						Lower bound	Upper bound
24 h after fabrication	Control	Air abrasion	−9.51*	0.19	0.000	−10.08	−8.94
		Acid etching	−6.54*	0.19	0.000	−7.11	−5.97
		Laser	−9.53*	0.19	0.000	−10.10	−8.96
	Air abrasion	Control	9.51*	0.19	0.000	8.94	10.08
		Acid etching	2.96*	0.19	0.000	2.39	3.53
		Laser	−0.025	0.19	1.000	−0.59	0.54
	Acid etching	Control	6.54*	0.19	0.000	5.97	7.11
		Air abrasion	−2.96*	0.19	0.000	−3.53	−2.39
		Laser	−2.99*	0.19	0.000	−3.56	−2.42
	Laser	Control	9.53*	0.19	0.000	8.96	10.10
		Air abrasion	0.025	0.19	1.000	−0.54	0.59
		Acid etching	2.99*	0.19	0.000	2.42	3.56
252 h	Control	Air abrasion	−9.32*	0.20	0.000	−9.92	−8.72
		Acid etching	−6.46*	0.20	0.000	−7.06	−5.86
		Laser	−9.44*	0.20	0.000	−10.03	−8.84
	Air abrasion	Control	9.322*	0.20	0.000	8.72	9.92
		Acid etching	2.856*	0.20	0.000	2.25	3.45
		Laser	−0.117	0.20	0.979	−0.717	0.482
	Acid etching	Control	6.46*	0.20	0.000	5.86	7.06
		Air abrasion	−2.85*	0.20	0.000	−3.45	−2.25
		Laser	−2.97*	0.20	0.000	−3.57	−2.37
	Laser	Control	9.44*	0.20	0.000	8.84	10.03
		Air abrasion	0.117	0.20	0.979	−0.48	0.71
		Acid etching	2.97*	0.20	0.000	2.37	3.57
504 h	Control	Air abrasion	−9.22*	0.21	0.000	−9.83	−8.60
		Acid etching	−6.41*	0.21	0.000	−7.02	−5.79
		Laser	−9.34*	0.21	0.000	−9.95	−8.72
	Air abrasion	Control	9.22*	0.21	0.000	8.60	9.83
		Acid etching	2.81*	0.21	0.000	2.19	3.42
		Laser	−0.118	0.21	0.980	−0.73	0.49
	Acid etching	Control	6.41*	0.21	0.000	5.79	7.02
		Air abrasion	−2.81*	0.21	0.000	−3.42	−2.19
		Laser	−2.93*	0.21	0.000	−3.54	−2.31
	Laser	Control	9.34*	0.21	0.000	8.72	9.95
		Air abrasion	0.118	0.21	0.980	−0.49	0.73
		Acid etching	2.93*	0.21	0.000	2.31	3.54
1,008 h	Control	Air abrasion	−8.97*	0.21	0.000	−9.59	−8.35
		Acid etching	−6.05*	0.21	0.000	−6.67	−5.42
		Laser	−9.11*	0.21	0.000	−9.73	−8.49
	Air abrasion	Control	8.97*	0.21	0.000	8.35	9.59
		Acid etching	2.92*	0.21	0.000	2.30	3.54
		Laser	−0.133	0.21	0.971	−0.75	0.48
	Acid etching	Control	6.050*	0.21	0.000	5.42	6.67
		Air abrasion	−2.92*	0.21	0.000	−3.54	−2.30
		Laser	−3.06*	0.21	0.000	−3.68	−2.44
	Laser	Control	9.11*	0.21	0.000	8.49	9.73
		Air abrasion	0.133	0.21	0.971	−0.48	0.75
		Acid etching	3.061*	0.21	0.000	2.44	3.68

* P<0.05 (significant)

**Figure 3:**
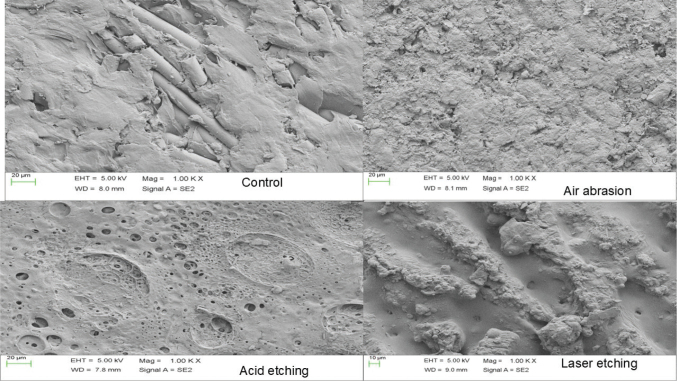
Scanning electron microscope image of PEEK after various surface treatment.

A scanning electron microscope (Carl Zeiss, Sigma-300, Gemini, Germany) was used to evaluate the surface topography of the PEEK specimen. Micro irregularities and cracks were found in untreated PEEK. There was an increase in surface irregularities and roughness in PEEK specimens after surface treatment with laser etching compared to air abrasion and sulfuric acid etching (Figure 3). The failure types observed are presented in Table 4. Laser etching, air abrasion, and sulfuric acid etching groups exhibited mostly cohesive and mixed failures, while the control groups exhibited only adhesive failure.

**Table 4:** Types of failures after shear bond strength test.

Types of failure	Control group	Air Abrasion group	Acid etch group	Laser treatment group
Adhesive	100.0%	20.0%	34.0%	23.5%
Cohesive	-	45.5%	39.5%	41.5%
Mixed	-	34.5%	26.5%	35.0%

## Discussion

The null hypothesis was rejected since the shear bond strength between PEEK and maxillofacial silicone after different surface treatments were significantly different. Furthermore, there were no significant variations in the shear bond strength between laser and air abrasion surface treatments. Shear bond strength test was done for evaluating the mechanical reliability of bonded materials such as in maxillofacial prosthetics and dental restorations.

PEEK is an alternative option for framework fabrication for a dental prosthesis. It is a polymer and needs to ensure high bond strength for the durability of the prostheses, so the surface of PEEK is treated with various agents and conditioned with accelerated ageing to simulate the clinical condition and to ensure the longevity of the prostheses. Fibre lasers are the newest advancements in laser technology, offering the benefits of efficiency, stability in oscillating modes, brilliance, and the potential for monolithic packaging. Fibre lasers are more potent and swifter than other lasers, and they cause less mechanical and thermal damage to surfaces [14].

To solve the difficulties of silicone elastomer delaminating from the retentive matrix, several possible solutions have been proposed. The potential use of fibre-reinforced composite (FRC) as a substructure material for facial deformity rehabilitation was evaluated by Kurunmäki and Kantola [15] and Dakshinamoorthy et al. [11] Fibre-reinforced composite is a material made up of inorganic glass fibres, fillers, and polymers. The shear bond between silicone and acrylic with different surface treatments was evaluated by Muhanad et al. [16], Hadded et al. [17], Rhea et al. [12], Faroouqi et al. [18], and Shetty et al. [19] The shear bond strength between PEEK and other materials like composite, acrylic and Co-Cr alloy with different surface treatments was evaluated by Caglar et al. [20], Sivaprakash et al. [6], Ahmet et al. [9], and Schmidlin et al. [21].

The effect of various surface treatments on the shear bond strength of PEEK and composite resin was investigated by Çulhaoğlu et al. [9] The maximum bond strength was obtained by acid-etched (98%H_2_SO_4_) PEEK surfaces, followed by laser-treated surfaces (Yb: PL laser), airborne particle abrasion (Al_2_O_3),_ and silica coating (CoJet). Caglar et al. [20] assessed how various surface treatments and adhesives affect the resin cement’s ability to adhere to PEEK. The values were higher for sandblasting (Al_2_O_3_), followed by silica coating (3 M ESPE, Seefeld, Germany) and laser treatment (Er: YAG) with adhesive application (Visio. Link). The efficacy of resin cement was unaffected by the 1.5 W (150 mJ) Er: YAG laser pretreatment of the PEEK surface.

The impact of 70%, 80%, 85%, 90%, and 98% sulfuric acid for 60 s on the shear bond strength of PEEK and resin composites was examined by Chaijareenont et al. [10] Shear bond strength was higher for PEEK surfaces pretreated with 90% and 98% sulfuric acid than for the other groups. The authors found that the primary determinant of the micromechanical bonding between PEEK and resin materials was surface topography.

Cevik et al. [22] concluded that the silicone primer (G611 platinum primer) alone significantly improved the bond between PEEK and silicone, while additional surface treatments had minimal impact. The results indicated that PEEK, especially when used with a primer, offers stronger bonding than PMMA and may be a more effective substructure material for implant-supported maxillofacial silicone prostheses. The impact of laser irradiation at a wavelength of 1,064 nm on the surface free energy of polymers was investigated by Wilson et al. [23] who found enhancement in surface free energy. Yilmaz et al. [24] demonstrated that the shear bond strength between PMMA and the CoCr alloy was higher following Nd: YAG laser irradiation of the sandblasted surface (Al_2_O_3_).

Ge et al. [25] conducted an *in vitro* analysis for 40 PEEK specimens that were randomly assigned to five groups: D-PEEK (treated with DMSO), B-PEEK (sodium borohydride), S-PEEK (98% sulfuric acid), and BS-PEEK (combined sulfuric acid and sodium borohydride). There was also a control group (P-PEEK). The findings showed that the B-PEEK, S-PEEK, and BS-PEEK groups had significant surface etching, while the S-PEEK and BS-PEEK groups had notable pore development. The strongest binding was shown by the BS-PEEK group. Combining sulfonation with NaBH₄ treatment (BS-PEEK) produced the best results, suggesting that this approach could be used to improve PEEK-silicone adhesion in maxillofacial prostheses.

PEEK and maxillofacial silicone form a micromechanical bond. PEEK samples without any surface treatment primarily displayed adhesive failure in the control group, whereas cohesive and mixed failure were observed in the air abrasion, acid etching, and laser etching group

This *in vitro* study revealed a statistically significant difference in shear bond strength between room temperature vulcanising maxillofacial silicone and PEEK treated with laser etching surface treatment. The superior bond found for both the laser and the air abrasion groups may be explained by the increased surface area created by roughness and micro irregularities. The same explanation may be applied for the sulphuric acid etch groups to a certain extent. Previous studies reported by Caglar et al. [20], Çulhaoğlu et al. [9], and Sivaprakash Babu et al. [6] confirm the results of the present study. In contrast, Yilmaz et al. [24], concluded that laser etching showed higher bond strength followed by sandblasting, while Cevik et al. [22] concluded that silicone primer showed better bond strength compared to laser etching.

PEEK is widely used in the medical and dental fields because of its unique properties. Its lightweight, polishable surface, colour stability, and low water absorption make it an excellent choice for fabricating various prostheses that are typically made from PMMA resin [26, 27]. In addition, PEEK’s ability to resist polymerisation shrinkage during manufacturing makes it a promising material for maxillofacial prosthetics, particularly as a substructure. Combining PEEK with 3D printing technology holds significant potential to advance and improve maxillofacial prosthetic fabrication [28].

A limitation of this study was the lack of uniformity of application of the laser etching, application being very difficult to standardise. Also, the silicone was mixed manually which may have led to inhomogeneity. Clinical research can also be performed to validate the results and assess the combined effects of mechanical and chemical processes on the bond strength between PEEK and maxillofacial silicone.

### Clinical significance

The findings of this study revealed that the highest shear bond strength between PEEK and maxillofacial silicone was achieved when the PEEK surface had been subjected to laser etching or air abrasion. Hence, laser etching may be considered as a preferred surface treatment to improve the interfacial durability and longevity of PEEK-based maxillofacial prostheses to improve the clinical outcomes and patient satisfaction.

## Conclusion

Within the limitations of study, the following conclusions were drawn:

1. Surface pretreatment of PEEK significantly influenced the adhesion of maxillofacial silicone to PEEK. Laser treatment and air abrasion provided superior and statistically comparable bond strengths, whereas sulfuric acid etching was less effective. Accelerated ageing reduced bond strength over time, highlighting the effect of ageing conditions on the durability of adhesion.
2. PEEK is a promising option to fabricate substructures for maxillofacial prostheses in combination with digital technologies to ensure precision and predictable outcome of prostheses, and laser treatment or air abrasion are recommended to ensure the overall success of the maxillofacial prostheses.
